# A dual pronged approach to cancer immune exclusion: tumor-derived LAIR-1 simultaneously drives fibrosis and blocks immune recruitment in glioma

**DOI:** 10.21203/rs.3.rs-10472790/v1

**Published:** 2026-08-05

**Authors:** Maria Luisa Varela, Henry Kast, Molly West, Marisa Rose, Daiana Perez Visñuk, Mary Violet Springer, Sadhakshi Raghuram, Ziwen Zhu, Yichen Wang, Brandon McClellan, Andrea Comba, Bhuvna Murthy, Fateme Karimi Hafshejani, Hend AlMunaidi, Marta Edwards, Joshua Welsh, Maria G. Castro, Pedro R. Lowenstein

**Affiliations:** 1 Department of Neurosurgery, University of Michigan Medical School; Ann Arbor, MI 48109, USA.; 2 Department of Cell and Developmental Biology, University of Michigan Medical School; Ann Arbor, MI 48109, USA.; 3 Department of Computational Medicine and Bioinformatics, University of Michigan; Ann Arbor, MI 48109, USA.; 4 Rogel Cancer Center, University of Michigan; Ann Arbor, MI 48109, USA; 5 Department of Biomedical Engineering, University of Michigan; Ann Arbor, MI 48109, USA

## Abstract

Glioblastoma is among the most lethal human malignancies. Immune-based therapies have failed due to a strong immunosuppressive tumor microenvironment (TME)^1^. We uncovered that LAIR-1^2^ expressed by tumor cells simultaneously drives TME fibrosis and inhibits migration of immune cells to brain tumors, thus achieving powerful immune exclusion^3^. We demonstrate that glioma-cell-specific LAIR-1 knockdown (KD), but not LAIR-1 KD from host cells, significantly extends survival in an immune-dependent manner. Glioma-cell-specific LAIR-1 signals through SHP2 to activate JNK –which on one hand sustains high levels of Lysyl Oxidase-Like 1 and collagen I to block immune cells’ entry– and on the other hand suppresses STAT3 signaling. In the absence of LAIR-1, gliomas’ collagen-dense ECM becomes disassembled and, through STAT3-driven upregulation of ADAM10 and ADAM17, promotes release of CXCL16, and recruitment of NK cells and cytotoxic T cells. Combining LAIR-1 KD with immune-stimulatory gene therapy^4^ achieved 100% long-term survival with durable immunological memory in immunocompetent mice. Pharmacological SHP2 inhibition in LAIR-1 WT mouse and human glioma cells recapitulated the LAIR-1 KD molecular phenotype and similarly potentiated gene therapy. These findings define LAIR-1 as a tumor-cell-intrinsic pro-fibrotic and immunosuppressive checkpoint and identify the LAIR-1>SHP2>JNK>CXCL16 and/or LOXL1 axis as a therapeutic target for sensitizing glioma to immunotherapy.

Immune exclusion from the tumor microenvironment (TME) is central to understanding the failure of immune therapies in gliomas and other tumors^1^. An inhibitory tumor microenvironment and paucity of intratumoral immune cells are two hurdles that need to be overcome to improve the efficacy of immunotherapies for solid tumors^3,5^. How these two phenomena are mechanistically linked is poorly understood. Here we show that LAIR-1^2^, expressed by glioma cells, acts as a collagen-activated signaling hub that sustains glioma fibrotic ECM architecture and suppresses cytokine driven tumor infiltration.

Our single cell RNA-seq data demonstrates expression of LAIR-1 by tumor cells^6^ in all glioma subtypes (**Sup Fig 1A-C**). In a similar manner, our genetically engineered mouse models (GEMMs)^7,8^ show LAIR-1 expression in glioma cells (**Sup Fig 1D**). Glioma-cell-intrinsic LAIR-1 was knocked down (KD) using Sleeping Beauty transposon-based somatic gene transfer across three independent GEMMs (Fig 1A), significantly extending survival (Fig 1B, **Sup Fig2**). LAIR-1 has also been described in CD45+ immune cells^2,9–15^(**Sup Fig 1F**). To test any contribution of LAIR-1 expression by the immune cells, we knocked down LAIR-1 from all host cells, including immune compartment. Importantly, when LAIR-1 was KD selectively in host cells mice bearing LAIR-1 wild type (WT) gliomas, there was no survival benefit in any of the three tumor models (**Sup Fig1E**). This highlights that the survival benefit is specifically attributable to loss of LAIR-1 in glioma cells and not in infiltrating immune cells or any other host-derived cell^9,16^.

Survival benefit in LAIR-1 KD conditions could be due to LAIR-1-induced changes in cell proliferation, or changes in the systemic immune response. Though a previous work postulated that these effects were mediated by changes in cell proliferation(**Sup Fig2C**)^6^, our work shows that the survival benefit of glioma-intrinsic LAIR-1 KD is entirely immune-dependent: the survival benefit was abrogated when LAIR-1 KD tumor cells were implanted into immunodeficient NSG mice, Nude mice, or into CD8-knock-out (CD8 KO) mice (median survival (MS) ~15–17 days across all immunodeficient groups vs 31 days in immunocompetent C57BL/6 controls (p<0.001; Fig 1C, **Sup Fig3**). This confirms that changes observed in cell proliferation are not responsible for the changes in survival.

We thus examined the extracellular matrix architecture (ECM) of LAIR-1 KD tumors. The ECM revealed a massively reduced reticulin staining (fibrillar collagen I and III) and immune-staining of collagen I (Fig 1 D–E). Immune infiltration of tumors was substantially improved. Tumors showed significantly increased densities of NK cells, CD8+ cytotoxic T cells, CD4+ helper T cells, CD20+ B cells, and CD11c+ dendritic cells, alongside a significant reduction in CD206+ M2-polarized macrophages (Fig 1F) among other changes (**Sup Fig 4**). LAIR-1 KD glioma cells implanted into Nude mice exhibited the same collagen I reduction as LAIR-1 KD glioma cells implanted in C57BL/6 mice, indicating that collagen loss is not secondary to altered immune cell content (**Sup Fig5A**). Together, these data establish that glioma-derived LAIR-1 drives ECM fibrosis and suppresses immune infiltration, and that its loss is sufficient to remodel the TME toward an immune-permissive state.

Our lab has developed the first-in-human gene therapy (GT) for adult high-grade glioma combining two adenoviral vectors encoding HSV1-thymidine kinase (Ad-TK, with systemic ganciclovir to induce immunogenic tumor cell death) and Flt3 ligand (Ad-Flt3L, to recruit and mature dendritic cells). This therapy safely induces tumor cell death and recruits functional dendritic cells to stimulate a targeted anti-glioma immune response^4^. We tested whether LAIR-1 KD could potentiate the activity of our immune-stimulatory GT (Fig1G–J, **Sup Fig6A**). While in mice implanted with WT tumors GT extended survival (MS: 21.5 days saline vs 33 days GT, **p=0.0035), in mice bearing LAIR-1 KD tumors GT induced a major quali- and quantitative improvement: all animals in the LAIR-1 KD + GT group achieved long-term survival (MS: undefined; 100% survival at day 90 follow-up), whereas LAIR-1 KD saline controls had an MS of 29 days (**p=0.0025)(Fig1H). Longitudinal bioluminescence imaging confirmed durable tumor clearance specifically in the LAIR-1 KD + GT group; tumor-derived flux fell to background levels immediately after GT and remained undetectable for at least 90 days (Fig1I). At 5 days post-GT, immunofluorescence showed CD8+ T cells densely concentrated at the tumor margins in LAIR-1 KD+ GT animals surrounding residual GFP+ tumor cells, in contrast to their sparse distribution in the WT tumors (Fig1J). Quantification of immune infiltration confirmed that LAIR-1 KD + GT animals had the highest intratumoral densities of NK1.1+ NK cells, Granzyme B + CD8+ cytotoxic T cells, Granzyme B+ CD4+ helper T cells, and Granzyme B+ B220+ cytotoxic B cells, of all four experimental groups (Fig1K–N). LAIR-1 KD tumors, both saline and GT-treated, contained significantly less collagen 1 in the ECM, when compared with LAIR-1 WT tumors, regardless of the treatment (**Fig Sup5B**). Thus, GT alone recruits a restricted immune cellular cytotoxic infiltrate that is insufficient for durable tumor clearance in the presence of collagen-dense LAIR-1 WT matrix, whereas the collagen-sparse matrix in the LAIR-1 KD tumors allows GT-induced cytotoxic infiltrates to expand and achieve tumor clearance (Fig 1K–N).

The long-term survivors from the LAIR-1 KD + GT group were subsequently rechallenged with LAIR-1 KD glioma cells; 50% were able to clear the tumor and become long-term survivors, while the other 50% exhibited extended MS, demonstrating the establishment of durable immunological memory (**p=0.0018, **Sup Fig6B**).

We determined the large-scale spatial organization of LAIR-1 WT and LAIR-1 KD tumors treated with GT using spatial proteomics. Neighborhood analysis of multiplex immunofluorescence identified three tumor compartments: immune-infiltrated tumor border, immune-infiltrated tumor, and non-infiltrated tumor (Fig 2A–B, **Sup Fig7–11**). In LAIR-1 WT tumors, non-infiltrated tumor area comprised 56% of the tumor, with a relatively limited immune-infiltrated zone (18%). GT increased the immune-infiltrated tumor area (45%) in LAIR-1 WT+ GT tumors. Further, the combination of LAIR-1 KD with GT produced a striking major reorganization: the immune-infiltrated tumor compartment expanded to 61% of the tumor area, while the non-infiltrated tumor shrank to 6% and the immune-infiltrated tumor border accounted for 33% (Fig2A–B, **Sup Fig11**). Cell-cell interaction analysis revealed that LAIR-1 KD tumors shifted tumor cells from a predominantly macrophage-centered interaction network (LAIR-1 WT) toward tumor-immune interactions and increased engagement of B220+, CD8+, CD4+ and DC populations, an effect most pronounced when combined with GT (Fig2C). Notably, LAIR-1 KD + GT group uniquely displayed repelling interactions between dendritic cells and T cells populations, reflecting organized immune network formation (Fig2C). Proximity density analysis further demonstrated that in LAIR-1 KD + GT tumors, dendritic cells closely associated with tumor cells, Granzyme B+ CD8+ T cells showed increased proximity to tumor cells, and CD8+ T cells were close to endothelial cells at the immune-infiltrated tumor border -consistent with active T cell extravasation-compared to LAIR-1 WT+ GT (Fig2G–I).

Spatial transcriptomics and CytoSignal ligand-receptor interaction analyses (Fig2D–F, **SupFig12**) revealed glioma-intrinsic LAIR-1 KD tumors have reduced contact-dependent collagen interactions, and diffusion-dependent TGF-β interactions, while diffusion-dependent CXCL16-CXCR6 interactions are enriched (Fig2E). Pathway enrichment analysis confirmed robust upregulation of lymphocyte activation terms (GO:0046649): lymphocyte proliferation, B cell activation, αβ T cell activation, and natural killer cell activation relative to LAIR-1 WT tumors (Fig2F).

The predominance of CXCL16-CXCR6-dependent interactions in LAIR-1 KD tumors prompted us to test the functional requirements for CXCR6-expressing effectors, i.e., NK and CD8+ T cells^17,18^, on tumor elimination when treated with GT. NK cells and CD8+ T cells were both independently necessary for increased survival: antibody-mediated NK cell depletion (αNK1.1)^19,20^, or implantation of LAIR-1 KD tumor cells into CD8 KO mice each reduced MS to approximately 21–26 days, abolishing the long-term survival phenotype (Fig3A–B).

To determine whether CXCL16-mediated immune recruitment is causally required for therapeutic synergy, we neutralized soluble CXCL16 with a blocking antibody in LAIR-1 KD + GT-treated mice (Fig3C). Anti-CXCL16 treatment abolished tumor regression (Fig3D), reduced MS to 21 days—identical to untreated WT controls—compared to undefined MS in IgG controls (p<0.001; FigE), and markedly reduced intratumoral NK1.1+, CD8+, and CD4+ T, and dendritic cells (Fig3F–I). Thus, CXCL16 is necessary component to generate anti-glioma response in LAIR-1 KD tumors.

To test if collagen I reduction in glioma ECM could be regulated by CXCL16, or the immune cells recruited by this cytokine, we analyzed the collagen I content in LAIR-1 KD + GT tumor treated with anti-CXCL16. There were no alterations in collagen I content (**Sup Fig13E**). Therefore, the regulation of collagen I in the ECM is regulated by glioma cells, and not immune cells.

We next dissected the molecular basis of LAIR-1’s dual control on ECM architecture and immune recruitment. Regarding ECM composition, LAIR-1 KD glioma cells expressed significantly less *Col1a1* and *Loxl1*, while they secreted more MMP-3 and MMP-9, and less TGF-β1 (Fig3J–N) when compared to LAIR-1 WT. Notably, reduced expression of Loxl1 was specific: other members of the lysyl oxidase family (*Lox, Loxl2, Loxl3, Loxl4*)^21^ were not reduced (**Sup Fig13A**), yet the total lysyl oxidase enzymatic activity was significantly reduced in LAIR-1 KD (Fig3L). LAIR-1 KD cells also showed increased levels of metallopeptidases *Adam10* and *Adam17*, which could explain LAIR-1 KD glioma conditioned medium containing markedly elevated soluble Adam10/17 shedding targets^22,23^: TNF-α, soluble CD40, soluble CXCL16 and CX3CL1 (Fig3O–Q, **Sup Fig13B-D**), when compared to LAIR-1 WT.

Acute LAIR-1 blockade by antibody neutralization in WT cells increased intracellular calcium activity (**Sup Fig14A-B**). The sustained elevation in intracellular calcium activity may contribute to increased cytokine secretion^24,25^ and/or shedding^26^, since calcium-influx is a well-established activator of ADAM10^27^. Consequently, we measured cytokines in LAIR-1 blockade conditioned media and found elevated NK-recruiting cytokines (soluble CXCL16, CXCL10, CX3CL1, CCL2) and NK-activating cytokines (IL-15, IL-12p40, IL-27, and TNF-α) and found them all (**Sup Fig14C-D**).

To dissect the signaling pathway downstream from LAIR-1, we examined the phosphorylation of LAIR-1’s proximal effector^28^. Western blot analysis determined LAIR-1 KD had reduced SHP2 phosphorylation (p-SHP2/t-SHP2), and when LAIR-1 WT cells are treated with the SHP inhibitor (NSC-87877) *Col1a1* and *Loxl1* expression and Lysyl oxidation activity were reduced (Fig4A–D). LAIR-1 KD also reduced JNK phosphorylation (p-JNK/t-JNK), and when LAIR-1 WT cells were treated with the JNK inhibitor (SP-600125) *Col1a1* and *Cxcl16* expression were also reduced (Fig4E–G). TFG β1 secretion was reduced following JNK inhibition (Fig4H). Finally, LAIR-1 KD had increased STAT3 phosphorylation (p-STAT3/t-STAT3), and when LAIR-1 KD cells were treated with the STAT3 inhibitor (Stattic), *Cxcl16, Adam17 and Mmp3* expression was reduced but, critically, *Col1a1* and *LoxL1* expression were not (Fig 4I–L, **Sup Fig15**).

These results define a LAIR-1 → SHP2→ JNK axis. Downstream of JNK two independent effector arms operate in parallel (Fig5). In the first arm, JNK drives *Col1a1* and *Loxl1* transcription, as well as TGF-β production. In the second arm, JNK-mediated suppression of STAT3 keeps *Adam10*, *Adam17*, and *Cxcl16* expression low, without altering collagen synthesis. JNK appears to act as a bifurcation node. JNK simultaneously engages both ECM remodeling and cytokine production/release.

In LAIR-1 WT tumor-bearing mice, SHPi administered concurrently with GT produced 100% long-term survival (MS: undefined), compared to MS of 20 days with saline(**p=0.01), 30.5 days with GT alone, and 21 days with SHPi alone (Fig4M–N), demonstrating that pharmacological SHP2 inhibition timed with GT is sufficient to convert a non-responsive tumor into a fully responsive one.

To test the role of human glioma cell intrinsic LAIR-1 we generated HF2303 human glioma cells^29^ with CRISPR-mediated LAIR-1 knock out (HF2303 LAIR-1 KO, **Sup Fig16**). These cells had reduced *COL1A1* and *LOXL1* expression, increased *CXCL16* expression and reduced TGF-β1 production (Fig 4O–R). HF2303 LAIR-1 KO confirmed that the regulatory axis is conserved in human glioma. In a similar manner, LAIR-1 WT HF2303 treated with SHP inhibitor elicited identical responses to those seen when LAIR-1 is knocked out (Fig 4S–V).

The role of tumor-cell LAIR-1 described here is mechanistically distinct from its function as inhibitory receptor in immune cells^2^, including tumor-associated macrophages^9,10^, and uncovers a new broad immune regulatory machinery: a SHP-JNK signaling branch (collagen I/LoxL1 and/or CXCL16) not captured by previous work^6^. Our data establish a **bifurcated** signaling axis downstream of collagen receptor LAIR-1 that simultaneously leads to a fibrotic ECM barrier and suppresses chemokine-driven immune recruitment. A single upstream node—JNK—coordinates both the construction of the collagen-dense barrier and the suppression of the signals that would recruit immune cells to the TME (Fig 5). In the first arm, JNK drives *Col1a1* and *LoxL1* transcription, sustaining fibrillar collagen deposition; in the second arm, JNK-mediated suppression of STAT3 keeps *Adam10*, and *Adam17* expression low, preventing proteolytic release of soluble CXCL16^30–32^. Disrupting this axis upstream of the bifurcation—by LAIR-1 KD or SHP2 inhibition—simultaneously collapses both arms, which explains the enhanced efficiency of the immune remodeling phenotype and the positive interaction with immune-stimulatory gene therapy.

The ECM phenotype regulation by glioma cells—collagen deposition and crosslinking via LOXL1^21,33^—was not characterized previously and constitutes a distinct, physical/biochemical mechanism of immune exclusion. Critically, our finding that conditional LAIR-1 KD restricted to host immune cells confers no survival benefit establishes that the tumor-cell compartment is the critical locus of LAIR-1 function.

Finally, we validate the conservation of this axis in human glioma using two orthogonal approaches —CRISPR-mediated LAIR-1 KO and pharmacological SHP2 inhibition— in HF2303 human glioma cells, demonstrating that reduced COL1A1 and LOXL1 expression, increased CXCL16 and reduced TGF-β1 production.

Glioma-cell intrinsic collagen I production correlates with malignancy^8^. In a similar manner, fibrosis remodeling is a recurrent driver of treatment resistance^34^. Here we describe a tumor-intrinsic driver of the fibrosis program, that is likely to be relevant for glioma treatments.

The spatial interaction data suggests that LAIR-1 targeted ECM remodeling is highly effective at reshaping the lymphocyte compartment. The LAIR-1 regulation of CXCL16 secretion is especially important regarding the increased efficacy of immune stimulatory gene therapy. Specifically, CXCR6^+^CD8^+^ T cells have been proposed to have tumor-antigen specificity and can enhance immune therapies^17,35^. If so, LAIR-1 KD would be recruiting a specific cytotoxic population with a particular relevance to tumor control, rather than a generic population.

Residual M2 macrophage activity may represent a resistance mechanism in tumors with high myeloid infiltration. The myeloid suppressive landscape in glioma extends beyond M2-polarized macrophages to include myeloid-derived suppressor cells (MDSCs), which are abundant in glioblastoma and suppress T cells and NK cells. Whether LAIR-1 loss additionally alters MDSC abundance, phenotype or suppressive function has not been directly assessed in our experiments and represents an important open question. The persistence of M2 macrophage-tumor cell attractive interactions in our most effectively treated animals suggests that residual myeloid immunosuppression may constitute a resistance mechanism in tumor with high myeloid infiltration such as GBM.

In summary, tumor-intrinsic LAIR-1 enforces a collagen-dense, CXCL16-restricted glioma microenvironment that excludes NK cells and cytotoxic lymphocytes and renders tumors refractory to immune-stimulatory gene therapy. A **bifurcated** JNK axis downstream of LAIR-1/SHP2 simultaneously maintains the physical ECM barrier through LOXL1-dependent collagen crosslinking and suppresses the chemokine gradient through STAT3-dependent repression of ADAM10/17. Disabling the LAIR-1/SHP2/JNK axis dismantles both barriers in parallel, generating a microenvironment in which immune-stimulatory gene therapy achieves complete, durable tumor control. These findings support the LAIR-1/SHP2/JNK axis as a combinatorial target in glioblastoma and establish a mechanistic framework for ECM-immune co-targeting as a strategy to sensitize immune-excluded tumors to immunotherapy.

## MATERIALS AND METHODS

### Animals

All animal experiments were performed in accordance with protocol PRO00012853 approved by the University Committee on Use and Care of Animals (UCUCA) at the University of Michigan. All animals were housed in an AAALAC accredited animal facility at the University of Michigan. Animals were monitored daily. All tumor bearing animals were euthanized at the time they developed clinical signs of tumor burden (impaired mobility, ruffled fur, hunched posture, protoporphyrin staining around eyes and nose, hunched posture, loss of coordination). Maximal tumor burden did not exceed IACUC guidelines.

### Genetically engineered mouse glioma models (GEMM)

We used genetically engineered mouse glioma models for survival analysis and histopathological analysis. Murine glioma tumors harboring different genetic drivers were generated using the Sleeping Beauty (SB) transposon system^1,2^. Genetic modifications were induced in postnatal day 1 (P01) male and female wild-type C57BL mice (Jackson Lab, stock #: 029567), according to UCUCA regulations.

To design and clone the shRNA targeting the *LAIR1* gene (pT2-shLAIR1-GFP4), we tested 3 22-base pair sequences for the mouse *LAIR1* gene selected from candidate sequences within the RNAi codex database (http://cancan.cshl.edu/cgi-bin/Codex/Codex.cgi) and InvivoGene (http://www.invivogen.com/sirnawizard). shLAIR1-A: AGTAAGACGCTGGAGCTAAAG, shLAIR1-B: GGCCAGTGAATGAAACCATTA, shLAIR1-C: TCTTATGATCTCCCAAGGGAC and shRNA Scramble: GAATCTAATCGTATCGTGGCTG. To confirm *LAIR1* knockdown, NIH/3T3 mouse cells were transfected with the designed shRNA plasmids for 72 h and subsequently analyzed by qPCR^3,4^.

Genetic models generated include the following gene expression/inhibitions: (i) ***N****RAS-G12V, sh****P****53*, and *sh****A****trx* (**NPA**), (ii) ***N****RAS-G12V* and *sh****P****53* (**NP**), (iii) ***N****RAS-G12V*, *sh****P****53*, and *P****d****gf*β (**NPD**), (iv) ***N****RAS-G12V*, *sh****P****53*, *sh****A****trx* and *sh****L****AIR1* (**NPAL**), (v) ***N****RAS*-*G12V*, *sh****P****53* and *sh****L****AIR1* (**NPL**) (vi) ***N****RAS-G12V*, *sh****P****53,P****d****gfβ*, sh***L****AIR1* (**NPDL**). Plasmid sequences were verified by Sanger sequencing. Plasmid sequences described below were used to generate tumors: (i) pT2C-LucPGK-SB100X, transposon & luciferase expression; (ii) pT2-NRAS-G12V, RTK/RAS/PI3K activation; (iii) pT2-shP53-GFP4, P53 knock-down; (iv) pT2-shAtrx-GFP4, ATRX knock-down; (v) pT2-shP53-Pdgfβ-GFP4, P53 knock-down in combination with PDGFβ ligand overexpression; (vi) pT2-shLAIR1-GFP4, LAIR-1 knock-down. Mice were injected with plasmids. Plasmid uptake in pups and tumor growth was monitored by IVIS^®^ Spectrum imaging. Adult mice displaying symptoms of morbidity were transcardially perfused^3^.

### Generation of primary mouse glioma neurospheres

Mouse glioma neurospheres were generated by harvesting brain tumors at the time of euthanasia by transcardial perfusion with Tyrode’s solution only. The brains were excised, and the tumors generated using the SB Transposon System were identified by GFP expression under an epifluorescence microscope at the time of resection. The tumor mass was dissociated using non-enzymatic cell dissociation buffer (BioLegend, Accutase, 423201), filtered through a 70 μm strainer and maintained in neural stem cell medium [DMEM/F12 with L-Glutamine (Gibco, 11320–033), B-27 supplement (Gibco, 12587–010), N-2 supplement (1X) (Gibco, 17502–048), penicillin streptomycin (100 U/mL) (Corning, 30–001-CI), and Normocin (InvivoGen, ant-nr-1)] at 37 °C, 5% CO_2_. FGF and EGF (Shenandoah Biotech, 100–26, 100–146) were added twice weekly at 6.6 ng/mL final concentration.

### Glioma cells and culture conditions

Mouse glioma cells (NPA, NPAL, NPD, NP, NPL and NPDL) were in DMEM/F12 with L-Glutamine (Gibco, 11320–033), B-27 supplement without Vitamin A (1X) (Gibco, 12587–010), N-2 supplement (Gibco, 17502–048), Penicillin-Streptomycin (10,000 IU Penicillin) (10,000 μg/mL Streptomycin) (Corning, Cellgro, 30–001-CI), and Normocin (Invivogen, ant-nr-1) at 37°C, 5% CO_2_, supplemented with EGF (Peprotech, 100–15) and FGF (Peprotech, 100–18) twice weekly at final concentration of 20 ng/mL.^1–5^, Human glioma cells (MGG8 and HF2303) were in DMEM with 10% Fetal bovine serum Glutamine (Gibco, A5256701), Penicillin-Streptomycin (10,000 IU Penicillin) (10,000 μg/mL Streptomycin) (Corning, Cellgro, 30–001-CI), and Normocin (Invivogen, ant-nr-1) at 37°C, 5% CO_2_^6^.

### Implantable murine glioma models: syngeneic and immune compromised models

For syngeneic immune competent models, we employed C57BL/6 mice (Jackson Lab, stock #: 029567). For total or partial immune compromised models, we employed: NOD SCID gamma (NSG) mice (inbreed in our facilities, from Jackson lab stock # 005557), CD8 knockout mice (inbreed in our facilities, from Jackson lab stock # 002665), or Nude mice (Jackson Lab, stock #:002019).

**Table:** 

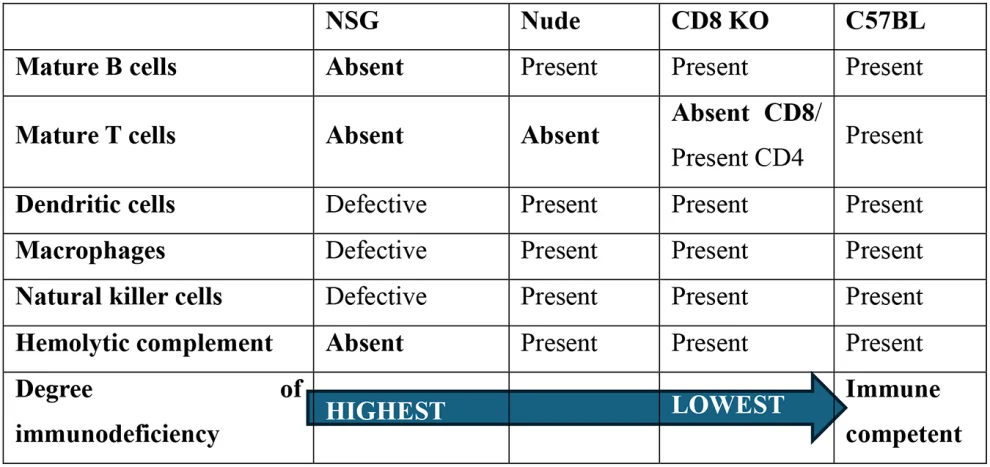

In every case, selected adult mice were stereotactic implanted with 3.0 × 10^4^ mouse glioma cells (NPA, NPAL, NP, NPL, NPD, or NPDL)^2–4,7^ into the mouse right striatum using a 22-gauge Hamilton syringe (1 μL/min) with the following coordinates: +1.00 mm anterior, 1.8 mm lateral, and 3.5 mm deep. The presence of tumors was verified 5 days post-implantation (DPI) by bioluminescence imaging.

### Gene Therapy

Two first-generation adenoviral vectors were used in this protocol: Ad.hCMV.hsFLT3L (Ad-Flt3L) + Ad.hCMV.TK (Ad-TK)^8^. Gene therapy was administered 7 and 9 days post LAIR-1 WT (NPA) and LAIR-1 KD (NPAL) tumor implantation, respectively, as described before^9^. Mice received 1 × 10^8^ plaque-forming units (pfu) of Ad-Flt3L and 2 × 10^8^ pfu of Ad-TK, or saline, in 1.5 μL volume in three locations at 3.5mm, 3.0mm, and 2.5mm ventral from the dura, followed by ganciclovir (GCV; TSZ Chemicals) administration twice a day for 7 days^7,8,10^.

### *In vivo* neutralizations: CXCL16 and NK1.1 depletion

For CXCL16 and NK1.1 depletion in mice, 200μg of *InVivo*MAb anti-mouse NK1.1 (Bio X Cell, BE0036) or 200ug of *InVivo*MAb anti-mouse CXCL16 (SR-PSOX) (Bio X Cell, BE0450), both diluted in *InVivo*Pure Dilution Buffer (Bio X Cell, IP0070), was injected intraperitoneally into tumor-bearing mice on the day prior to adenoviral administration and days 3, 7, and 11 after.

### SHP inhibition

For SHP inhibition *in vivo*, 375 μg of NSC-87877 (MedChemExpress, HY-18756), diluted in PBS, was injected intraperitoneally into tumor-bearing mice daily from days 8–14 post tumor implantation. For SHP inhibition *in vitro*, mouse cell lines were treated with 100 μM NSC-87877 and human cell lines were treated with 200 μM NSC-87877 then harvested 48 hours after treatment.

### LAIR1 inhibition

For LAIR1 inhibition *in vitro*, mouse cell lines were treated with 2.5 μg/mL of Recombinant Mouse LAIR1 Fc Chimera Protein (R&D Systems, 10–092-LR050) and human cell lines were treated with 2.5 μg/1mL of Recombinant Human LAIR1 Fc Chimera Protein (R&D Systems, 99–64LR-050) then harvested 48 hours after treatment.

### Immunohistochemistry on paraffin embedded brain tumors (IHC-DAB)

Following perfusion with Tyrode saline solution, mouse brains were fixed in 4% paraformaldehyde (PFA), and post-fixed for an additional 48 h at 4 °C. Brains were then processed in paraffin at the University of Michigan Orthopaedic Research Laboratories Histology Core and subsequently embedded in paraffin using Tanner Scientific TN1700 Embedding Center. Tissue was sectioned using a rotary microtome (Leica) set to 5μm in the *z*-direction. Endogenous Peroxidase quenching was carried out using a 0.3% H_2_O_2_ incubation for 5 min at room temperature. Heat-induced antigen retrieval was performed using 10 mM citric acid, 0.05% Tween 20, pH 6.0. Tissue permeabilization and blocking were completed using PBS 0.2% Tween-20 with 5% goat serum for one hour at room temperature. Tissue was incubated with primary antibodies at 4 °C overnight at concentrations detailed in Supplementary Table 1. Tissue sections were then incubated with secondary biotinylated antibodies (Polyclonal Goat Anti-Rabbit Immunoglobulins/Biotin, Agilent (E043201–6) at a 1:1000 dilution in PBS with 0.2% Tween-20 overnight at 4 °C. ABC Avidin-Biotin-Complex Binding reagent (Vectastain Elite ABC kit) and Betazoid DAB Chromogen detection kit (BioCare BDB2004) was used according to the manufacturer’s instructions.

### Immunofluorescence on paraffin-embedded sections from brain tumors

Following perfusion with Tyrode saline solution, mouse brains were fixed in 4% paraformaldehyde (PFA), and post-fixed for an additional 48 h at 4 °C. Brains were then processed in paraffin at the University of Michigan Orthopaedic Research Laboratories Histology Core and subsequently embedded in paraffin using Tanner Scientific TN1700 Embedding Center. Tissue was sectioned using a rotary microtome (Leica) set to 5 μm in the *z*-direction. Permeabilization was performed using 0.5% TritonX-100 in PBS for 30 min at room temperature while shaking. Heat-induced antigen retrieval was performed using 10 mM Citric Acid, 0.05% Tween 20, pH 6.2. Tissue sections were blocked in 10% horse serum and 3% BSA in PBS. This method was performed following previous protocol^3,4^. Primary antibodies were incubated at 4 °C overnight in a humid chamber with 5% horse serum in PBS at concentration detailed in Sup Table 1. Tissues were then incubated overnight at 4 °C with fluorescently labeled conjugated secondary antibodies at a dilution of 1:1000 with 3% BSA in PBS. Secondary antibodies names and used concentrations are detailed in Supplementary Table 1. After washing with PBS 6 times, 5 minutes each, nuclei were stained with DAPI (Invitrogen, D3571) for 5 minutes. Slides were washed with PBS and mounted with ProLong Gold Antifade Mountant (Invitrogen, P36930). Images were captured using a single photon laser scanning inverted confocal microscope LSM 880 AxioObserver (Carl Zeiss, Jena, Germany). Multichannel immunofluorescence images were analyzed using QuPath^11^.

### Western Blot

For western blotting, whole-cell lysates were prepared by resuspending cells in RIPA lysis buffer with 1x of Halt protease and phosphatase inhibitor cocktail (Thermo Fisher Scientific, 89900). 20 μg of protein extract (determined by bicinchoninic acid assay (BCA), Pierce, 23227) were separated by 4–12% SDS-PAGE (Thermo Fisher Scientific, NuPAGE, NP0322BOX) and transferred to nitrocellulose membranes (Bio-Rad, 1620112). Blocked in 5% BSA in TBS, probed with primary antibodies at the indicated dilutions (Supplementary Table 1) and detected with horseradish-peroxidase-linked anti-rabbit IgG (Cell Signaling Technology, 7074) or anti-mouse IgG (Cell Signaling Technology, 7076). Blots were imaged using a ChemiDoc MP imaging system (Bio-Rad).

### Cytokine array

To measure secreted cytokines, concentration-normalized cell culture supernatant samples were generated using Pierce^™^ BCA Protein Assay Kit with respective cell lysates (Thermo Scientific, 23227). Immunoblotting was performed using the Proteome Profiler Mouse XL Cytokine Array kit (R&D Systems, ARY028) following the manufacturer’s instructions. Immunoblots were imaged using SuperSignal^™^ West Femto Maximum Sensitivity Substrate (Thermo Scientific, 34096) in a ChemiDoc MP Imaging System (Bio-Rad). Immunoblots were analyzed in ImageJ2 using the protein array analysis tool.

### RNA isolation and RT–qPCR

Total RNA was isolated using the RNeasy Plus Mini Kit (QIAGEN, 74136) following the manufacturer’s instructions, and concentration was quantified using nanodrop (Thermo Fisher Scientific). cDNA was synthesized using 1μg extracted RNA with the SuperScript^™^ VILO^™^ cDNA Synthesis Kit (Invitrogen, 11754250) following the manufacturer’s instructions. qPCR was conducted using Fast SYBR^™^ Green Master Mix (Applied Biosystems, 4385612) and analyzed using QuantStudio^™^ 3 Real-Time PCR System (Applied Biosystems). PCR conditions are 50°C for 2 min, 95°C for 10 minutes, followed by 40 cycle amplification (95 °C for 15 s, 60 °C for 1minute). Relative mRNA expression was determined by normalizing to 18s expression, which served as an internal control.

### Reticulin Staining

Reticulin stain was done using Reticulum Stain Kit (Modified Gomori’s) (Abcam, ab236473) following the manufacturer’s instructions.

### Lysyl Oxidase Assay

Lysyl oxidase enzymatic activity was measured using the Lysyl Oxidase Activity Assay Kit (Abcam, ab284573) following the manufacturer’s instructions. Cell media was concentrated using 10k Da spin columns (Abcam, ab93349).

### Cell Proliferation Assay

Cell proliferation was measured using CellTiter-Glo(R) 2.0 Viability Assay (Promega, G9242) following the manufacturer’s instructions

### RNA Scope

RNAscope in-situ hybridization was performed on 5-μm coronal cryosections mounted onto positively charged slides, following the manufacturer’s protocol (ACDBio). Custom-designed probes targeting *LAIR1* and *GFP* mRNA (ACDBio) were used. Antigen retrieval was carried out to enhance probe penetration and signal amplification, followed by hybridization and detection using the RNAscope Multiplex Fluorescent v2 Assay Kits. Sections were counterstained with DAPI (ACDBio) and either imaged immediately or stored overnight at 4 °C in the dark to minimize photobleaching. Images were captured using a single photon laser scanning inverted confocal microscope LSM 880 AxioObserver (Carl Zeiss, Jena, Germany).

### Spatial proteomics

Multiplex immunofluorescence of mouse FFPE tissue was performed on a PhenoCycler-Fusion by Akoya Biosciences per manufacturer’s instructions with the following addition. After antigen retrieval, a photobleaching protocol was performed to reduce autofluorescence. Briefly, slides were submerged in a solution of 24 mM NaOH, 4.5% H_2_O_2_ in PBS, and sandwiched between two full spectrum LED light boxes for 45 minutes. Samples were then equilibrated in four three-minute PBS washes before continuing with the remainder of the Akoya protocol for FFPE tissue. Akoya antibodies, listed in the materials section, were used, except for a fraction that were custom conjugated to Akoya barcodes. For the Akoya protocol, samples are stained with antibodies conjugated to unique oligonucleotide sequences (i.e. barcodes). Reporters, consisting of fluorophores attached to oligonucleotide sequences complementary to those on the barcodes, are hybridized and imaged three at a time in consecutive cycles on the Phenocycler-Fusion platform.

The QPTIFF images generated were imported into QuPath version 0.5.0^11^ for sample annotation, cell segmentation, and cell phenotyping. Samples were annotated to include the tumor bulk, identified by GFP positivity, as well as the peritumoral area. The peritumoral area was defined as a 250 μm radius expansion of the tumor bulk annotation for all samples. Next, areas with artifacts were excluded. Segmentation was performed on the final annotation using the StarDist segmentation script^12^. Approximately 792,000 total cells from 12 samples were carried through to further analysis. After segmentation, phenotyping was performed using a hierarchical scheme implemented with a script using object classifiers that were trained using artificial neural networks (Sup Fig. 7A).

After classification of all cells was complete, data was exported into CytoMAP^13^ for neighborhood analysis. Raster scanned neighborhoods with a 50 μm radius were defined. Resulting neighborhoods were clustered using the NN self organizing map algorithm for clustering neighborhoods into **four** regions: (i) Enriched in tumor cells with low proportion of immune cells, named “**non-infiltrated tumor”** region; (ii) Enriched in tumor cells and immune cells, named “**immune-infiltrated tumor**” region; (iii) Anatomically located at the peritumoral area, with low proportion of tumor cells and enriched in immune cells, named “**immune-infiltrated tumor border**” region; (iv) Anatomically located at the peritumoral area, with low proportion of tumor cells and immune cells, named “**non-infiltrated tumor border**” region. The non-infiltrated tumor border was excluded from the analysis, dut its lack of both tumor cells and immune cells. The three remaining regions were analyzed in Scimap. The sm.tl.spatial interaction function with neighbor determination by radius from a cell centroid was utilized for cell interactions. The sm.tl.spatial pscore function was used for the determination of proximity density between specific cell types.

### Spatial transcriptomics

Tissue preparation and H&E staining were performed per the Visium HD (10x Genomics) FFPE Tissue Preparation Handbook (CG000684). H&E imaging, sample processing for the Visium HD workflow, library preparation, and next-generation sequencing were carried out by the Advanced Genomics Core at the University of Michigan. SpaceRanger v4.0 was utilized to align reads and generate feature matrices, as well as for cell segmentation and clustering. Cell segmentation was done using default parameters except that --nucleus-expansion-distance-micron was set to 5 for improved clustering of immune cell groups. Loupe Browser v9.0.0 was used to visualize gene expression and clustering of 8 μm bins, as well as for the re-analysis of the tumor bulk area, with cell density used to delineate the bulk tumor border. Marker gene analysis and the web-based gene list enrichment analysis tool, Enrichr^14^, were used to assign broad cell phenotypes for downstream analysis. For the dot plots, g:Profiler^15^was utilized to obtain GO Biological Process terms associated with each cluster, and the top 20 terms under the GO parent term “lymphocyte activation” (GO:0046649) were visualized after simplifying the immune-branch results using the “Rel” measure via clusterProfiler::simplify in R.

### Ligand-receptor analysis

We used CytoSignal v 0.5.0^16^ ligand-receptor co-expression inference in the spatial transcriptomics data. The LAIR-1 WT and KD slices were subset to the tumor region. We used the 16-micron-binned expression matrix produced by 10X space ranger to quantify the ligand-receptor local co-expression score (LRscore) individually for each of the two slices. We used an expanded database beyond the CytoSignal’s built-in resource, referring to CellPhoneDB v5^17^, to assess the signaling of pathways of interests but not available in CytoSignal. We further combined the LAIR-1 WT and KD slices together and used the CytoSignal downstream analysis function runNEBULA^18^(Nebula v1.5.3) to detect differentially signaling interactions between the two conditions. The score of a pathway in each spatial bin was defined as the mean of LRscores of all the interactions belonging to the pathway.

### ELISA

TGF-β ELISA was performed using Human/Mouse/Rat/Porcine/Canine TGF-beta 1 Quantikine ELISA (R&D Systems, DB100C) with cell supernatant following the manufacturer’s instructions.

### Human Cell LAIR-1 Knockout

LAIR1 Knockout was achieved using the LAIR-1 Double Nickase Plasmid (Santa Cruz Bio., sc-403542-NIC) and using the marching Control Double Nickase Plasmid (Santa Cruz Bio., sc-437281). Cells were transfected upon reaching 60% confluency with 2μg of plasmid and 10μL of transfection reagent. Cells were expanded to a larger vessel 72 hours post transfection and then allowed to grow for an additional 48 hours before being selected for with 2.5μg/mL puromycin antibiotic. Selection verified by fluorescent microscopy. Cells assayed after selection had been completed.

### Calcium Activity

NPA (NRAS/shP53/shATRX) glioma cells were infected with LV-CAG-GCaMP6f (SignaGen Laboratories, SL100321) reporter to enable live-cell calcium-associated fluorescence imaging. GCaMP6f-expressing glioma cells were seeded into two-chamber cover glass slides (Thermo Scientific^™^ Lab-Tek^™^ II Chambered Coverglass, cat. no. 155379) and imaged by confocal microscopy under control or LAIR1 inhibitor-treated conditions. LAIR1 inhibitor was used at a final concentration of 5μg/mL. Time-lapse movies were acquired as single-channel image sequences with an interval of 615.12 s per frame, 122 total frames, and a pixel size of 0.4151329 μm/pixel.

Calcium-associated fluorescence was analyzed using a custom Python pipeline. For each movie, a global fluorescence mask was generated from the maximum intensity projection using Gaussian smoothing, Otsu thresholding, morphological opening, and size filtering. Background fluorescence was estimated for each frame from the lower 5th percentile of pixel intensities and subtracted before fluorescence quantification. Mean background-corrected fluorescence was calculated within the global mask for each frame. Fluorescence was normalized to each movie’s baseline and expressed as ΔF/F_0_, where F_0_ was defined as the 10th percentile of the corrected fluorescence trace.

For the calcium analysis, mean ΔF/F_0_ was calculated for each movie during the 250–600 min late-phase analysis window. Each movie was treated as one independent replicate. Control and LAIR inhibitor-treated groups were compared using Welch’s t-test, and effect size was calculated using Cohen’s d. Time-course data were plotted as mean ± SEM from 0 to 600 min, and exploratory time-point comparisons were performed using Welch’s t-test with Benjamini–Hochberg false discovery rate correction for multiple comparisons.

### Statistical analysis

All quantitative data are presented as the means ± SEM from at least three independent samples. ANOVA and two-sample *t* tests were used to compare continuous outcomes between groups. Survival curves were analyzed using the Kaplan-Meier method and compared using Mantel-Cox tests; the effect size is expressed as MS. Differences were considered significant if *P* < 0.05. All analyses were conducted using GraphPad Prism software (version 11.00) or R (version 3.4). The statistical tests used are indicated in each figure legend.

## Supplementary Material

Supplementary Files

This is a list of supplementary files associated with this preprint. Click to download.

- SIFiguresvarelaetal2026.pdf

## Figures and Tables

**Figure 1.**
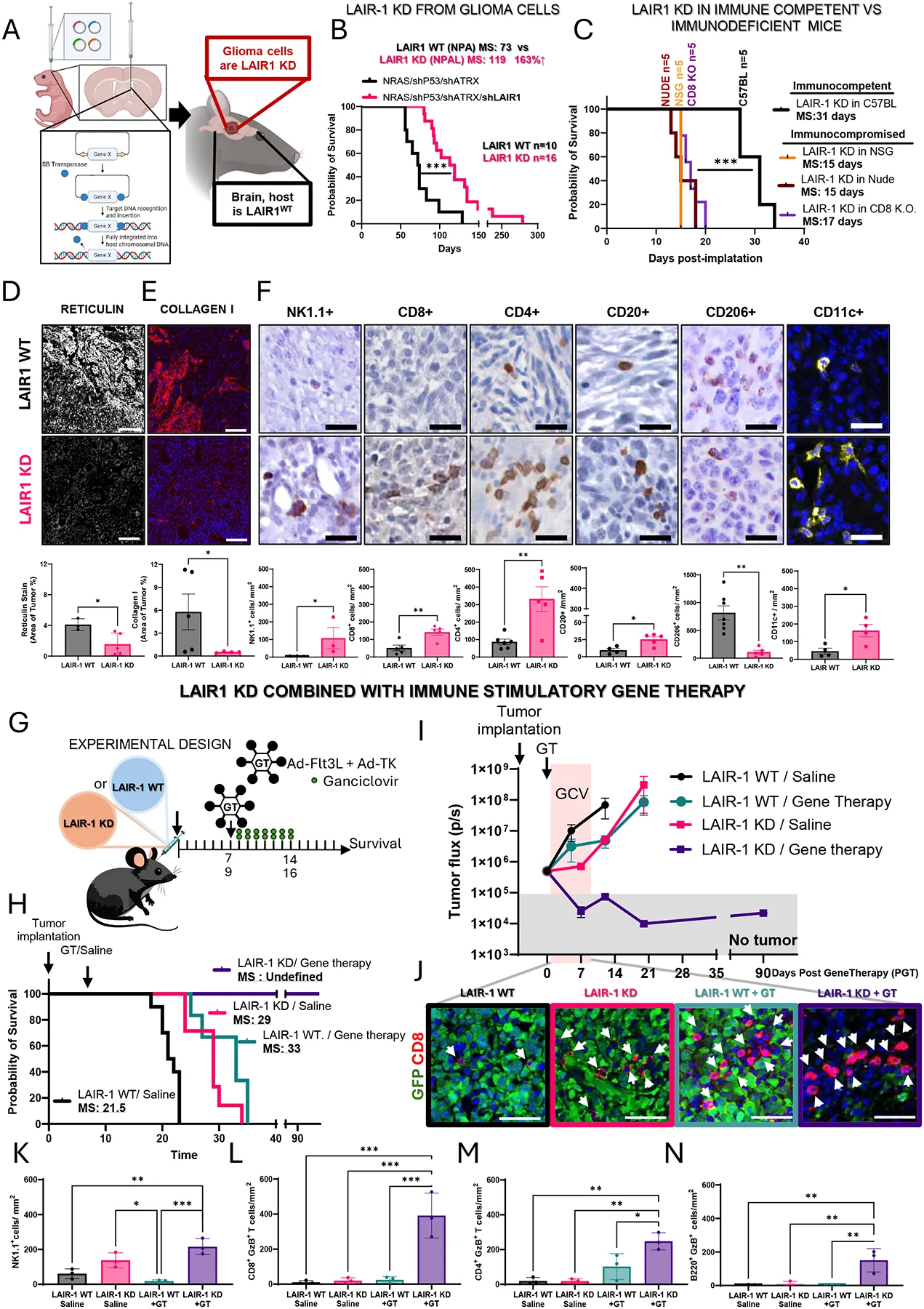
**A.** Schematic of the establishment of genetically engineered mouse models (GEMMs) with LAIR-1 downregulation in glioma cells. One-day-old LAIR-1 wild type pups are injected with plasmids coding for driver glioma mutations to be expressed only within stem cells lining the lateral ventricle. These mutations are integrated into the genome by the Sleeping Beauty (SB) transposon system. By adding a plasmid coding for LAIR-1 shRNA we generated glioma cells with LAIR-1 knockdown, thus obtaining a LAIR-1 knockdown glioma within a wild type host. **B.** Survival curve of mice bearing LAIR-1 knockdown (KD) glioma tumors. Median survival (MS) of the LAIR-1 KD group (●) was 113 days, significantly longer than the LAIR-1 Wild Type (WT) (●) group (MS: 73 days). Mantel-Cox test, ***p= 0.0004. **C.** Survival curve of immune competent and partial or total immuno-compromised mice implanted with LAIR-1 KD glioma cells. The survival increase of the LAIR-1 KD group is lost in immune suppressed animals (NSG, Nude and CD8 KO mice); Mantel-Cox test, C57BL/6 vs NSG (***p= 0.0027), vs Nude (***p= 0.0027), vs CD8 KO (**p= 0.0008); N=5/group. **D.** Detection of collagen I and III fibers within the extracellular matrix by reticulin staining in LAIR-1 KD and WT brain tumor sections. Quantification (shown for all conditions below the histology) showed the reticulin positive % of tumor area is decreased in LAIR-1 KD tumors; Mann Whitney test, *p= 0.0357. Scale bar is 100 μm. **E.** Collagen I (red) staining by immunofluorescence within the extracellular matrix in LAIR-1 KD and WT brain tumor sections. Quantification shows collagen I positive % of tumor area Mann Whitney test, *p=0.0317. Nuclei in blue (DAPI). Scale bar is 100 μm. **F.** Immunohistochemistry identification of immune infiltrating cells in LAIR-1 KD and WT brain tumor sections. The following antibodies were used to identify immune cells: NK1.1 to identify NK cells(*p=0.0201), CD8 to identify cytotoxic T cells (**p=0.0053), CD4 to identify helper T cells(**p=0.0048), CD20 to identify B cells (*p=0.0134), CD206 to identify M2 macrophages(**p=0.0010) and CD11c to identify Dendritic cells(*p=0.0236). Quantification shows positive cells per mm^2^. Unpaired test. Scale bar is 25 μm. All immune cells were increased in LAIR-1 KD tumors; only M2 macrophages were decreased. **G.** Experimental design schematic. Adult C57B mice were implanted with LAIR-1 KD or LAIR-1 WT glioma cells (day 0). When tumors reached 5×10^5^ flux (photos/sec) (~day 7 for LAIR-1 WT and day 9 for LAIR-1 KD, post tumor implantation) animals received gene therapy (GT): intratumoral administration of 2 adenoviral vectors (Ad-Flt3L and Ad-TK) or saline control, followed by 7 days twice daily i.p. administration of ganciclovir. **H.** Survival curve of LAIR-1 KD and WT treated with or without GT, as described in **G**. GT increased MS in LAIR-1 WT (●) from 21.5 to 33 days(***p=0.0004), and in LAIR-1 KD (●) from 29 days to undefined (100% long-term survivors)(***p=0.0009). Mantel-Cox test. **I.** *In vivo* tumor size monitoring. Flux <9×10^4^ indicates absence of tumor. Tumor follow up continued until day 90 for the survivors (LAIR-1 KD + GT). **J.** Immunofluorescence detection of tumor cells (GFP (green)) and T cells CD8 (red) in brain sections of mice 5 days post GT. White arrows show CD8^+^ cells infiltrating the tumors. Note the progressive increase in CD8+ T cells; in the LAIR-1 KD + GT group, T cells are surrounding the few remaining tumor cells. Scale bar is 50 μm. **K-N.** Number of immune cells infiltrating the tumor area detected by standard or multiplex immunofluorescence in LAIR-1 WT (●), LAIR-1 KD (●), LAIR-1 WT + GT (●) and LAIR-1 KD+ GT (●) brain sections. **K.** NK cells (NK1.1^+^ cells). **L.** Granzyme B^+^ cytotoxic T cells (CD8^+^ and GzB^+^ cells). **M.** Granzyme B^+^ helper T cells (CD4^+^ and GzB^+^ cells). **N.** Granzyme B^+^ B220^+^ cells (B220^+^ and GzB^+^ cells). All immune cells studied display a large increase in LAIR-1 KD + GT animals. *p<0.05, **p<0.01, ***p<0.001. Bar graphs show mean and SEM. One-way ANOVA and Tuckey test.

**Figure 2.**
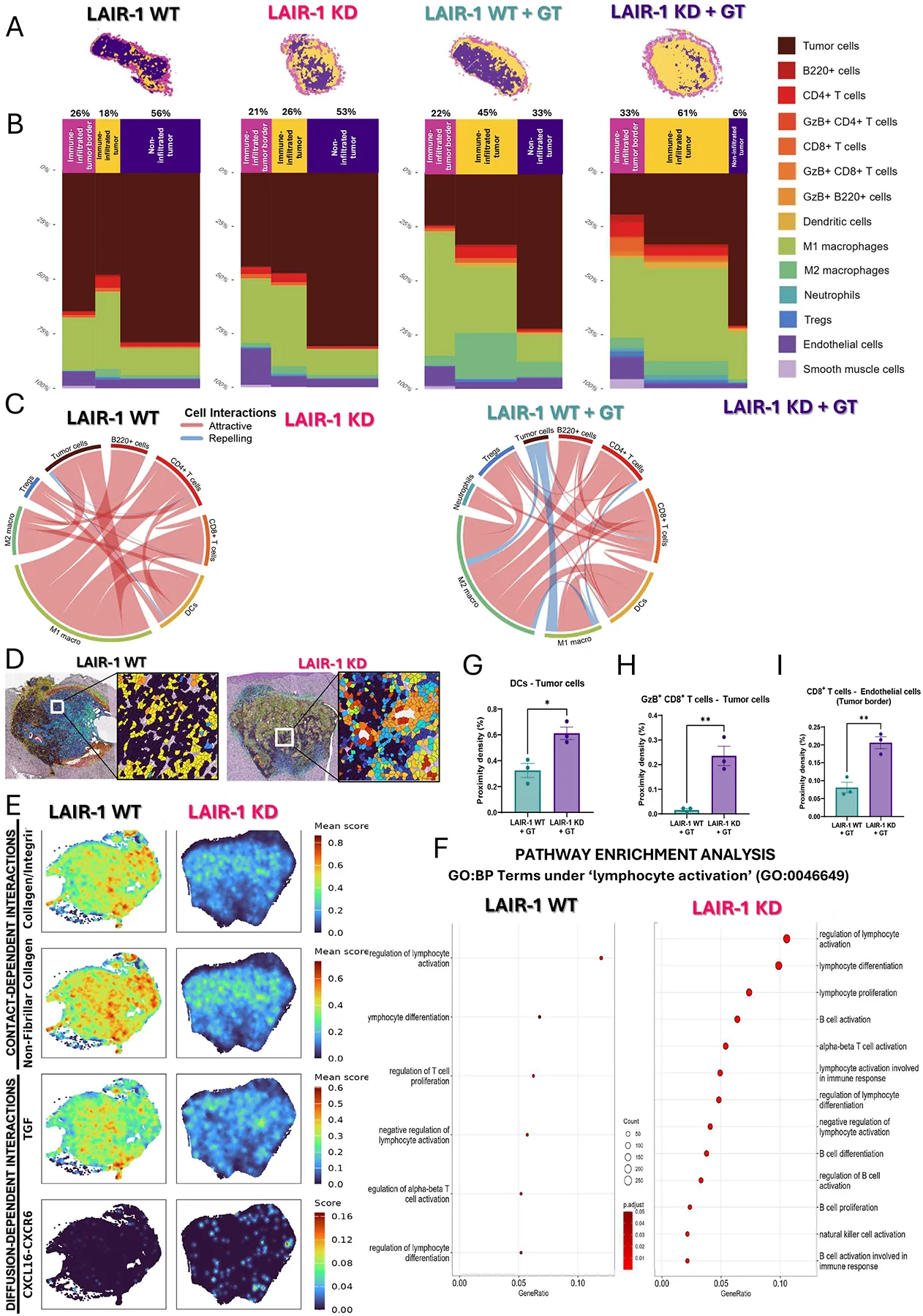
**A.** Representative neighborhood maps from LAIR-1 WT and KD glioma samples, treated with and without GT, illustrating the spatial distribution of cellular neighbourhoods within each tumor: immune-infiltrated tumor border, immune-infiltrated tumor, and non-infiltrated tumor. **B.** Prevalence (%) of each neighborhood is shown at the top. Cellular composition of each neighborhood is shown below neighborhood name. Notice the massive increase of the immune infiltrated tumor neighborhood in LAIR-1 KD + GT. **C.** Chord diagrams of attractive (red) and repelling (blue) cell interactions between major cell types. The width of each chord reflects the strength of the interaction. **D-F.** Visium HD spatial transcriptomic analysis of WT and LAIR-1 KD tumors. **D.** Tumor region after annotation, cell segmentation, and graph-based clustering (Louvain method). **E.** CytoSignal ligand-receptor local co-expression score (LRscore) in each spatial bin demonstrated for either LAIR-1 WT or LAIR-1 KD tumors. Collagen and TGFβ interactions are shown for all possible binding partners. **F.** Dot plot of significant GO biological process terms associated with the parent term ‘lymphocyte activation’ (GO: 0046649) obtained via pathway enrichment analysis from the major immune cluster in each sample. **G-I.** Proximity density graphs showing the proportion of defined interactions relative to all interactions between the corresponding cell types in LAIR-1 WT + GT vs LAIR-1 KD + GT glioma samples. **G.** Dendritic cells (DCs) to tumor cells (*p=0.0164). **H.** Granzyme B ^+^CD8^+^ T cells to tumor cells (**p=0.0051). **I.** CD8^+^ T cells to endothelial cells, in the tumor border compartment (**p=0.0051). Note how the TME becomes progressively more immune rich as LAIR-1 KD tumors are treated with GT. Bar graphs show mean and SEM. Unpaired t test.

**Figure 3.**
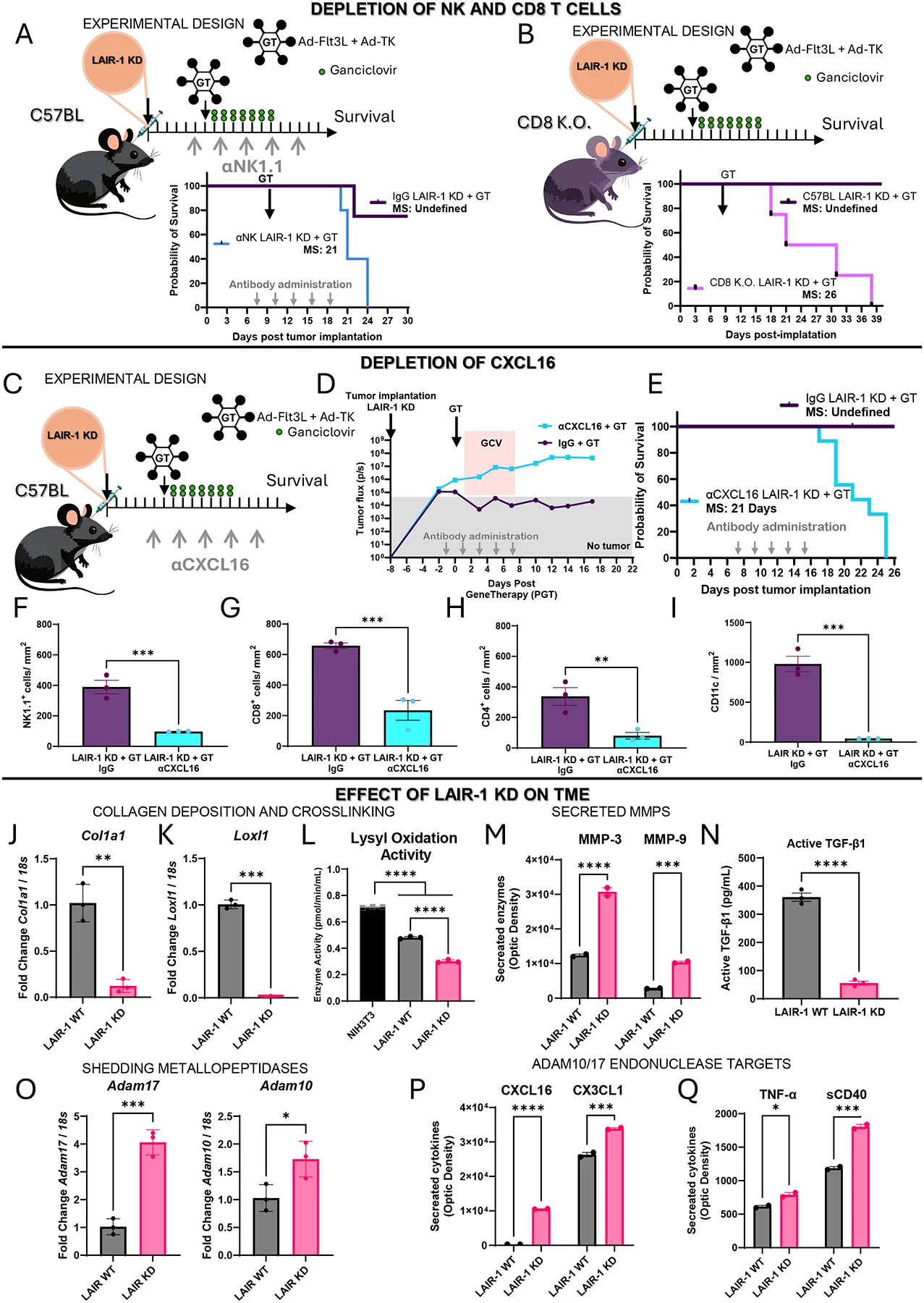
**A-B. Depletion of CXCR6+ effector cells. A.** Adult C57BL mice were implanted with LAIR-1 KD glioma cells (day 0). When tumors reached 5×10^5^ flux photos/sec animals received GT: i.t. Ad-Flt3L and Ad-TK, or saline control, followed by 7 days of ganciclovir twice daily i.p. One day before GT administration and every 3 days animals were injected with antibodies against NK1.1(CD161) to deplete NK cells (●), or control IgG (●), and survival was registered. LAIR-1 KD + GT has undefined MS. The LAIR-1 KD + GT + NK depletion group has MS:21 days, having lost the ability to potentiate GT. (Mantel-Cox, **p= 0.0393). **B.** Adult CD8 KO (●) or C57BL (●) mice were implanted with LAIR-1 KD glioma cells (day 0). When tumors reach 5×10^5^ flux photos/sec animals receive GT: i.t. Ad-Flt3L and Ad-TK, or saline control, followed by 7 days of ganciclovir twice daily i.p. LAIR-1 KD + GT in C57BL mice had undefined MS. While LAIR-1 KD + GT in CD8 KO mice had MS:26 days, having lost the ability to potentiate GT. (Mantel-Cox, **p= 0.0027). **C-I. Depletion of CXCL16. C.** Experimental design schematic. Adult C57BL mice were implanted with LAIR-1 KD glioma cells (day 0). When tumors reached 5×10^5^ flux photos/sec animals received GT: i.t. Ad-Flt3L and Ad-TK, or saline control, followed by 7 days of ganciclovir twice daily i.p. One day before GT administration and every 3 days animals were injected with antibodies against CXCL16 (αCXCL16) (●), or control IgG (●); and survival was registered. **D.** *In vivo* tumor size monitoring. Flux <9×10^4^ is considered background luminescence levels indicating tumor regression. **E.** Survival curve of LAIR-1 KD + GT treated with αCXCL16 or IgG. LAIR-1 KD + GT + IgG group has undefined MS, while CXCL16 neutralized group has MS:21 days, having lost the ability to potentiate GT. (Mantel-Cox, **p= 0.0038) **F-I.** Number of immune cells infiltrating the tumor area detected by immunofluorescence in in LAIR-1 KD + GT + IgG and LAIR-1 KD + GT + αCXCL16. **F.** NK cells (CD161^+^ cells) (**p= 0.0027) **G.** Cytotoxic T cells (CD8^+^ cells) (**p= 0.0033) **H.** Helper T cells (CD4^+^ cells) (*p= 0.0146). **I.** Dendritic cells (CD11c^+^ cells) (***p= 0.0007). Bar graphs show mean and SEM. Unpaired t test. **J-Q. Effect of LAIR-1 KD on TME. J-L.** Fold change of *Col1a1***(J)** and *Loxl1* **(K)** *in vitro* gene expression of LAIR-1 WT and LAIR-1 KD cells by qPCR, relative to 18s expression. **L.** Lysyl Oxidation Activity detected *in vitro* in cell supernatants of LAIR-1 WT, LAIR-1 KD and positive control (mouse fibroblasts (NIH3T3 cells)). **M.** Metalloproteases *in vitro* secretion (MMP-3 and MMP9) LAIR-1 WT and LAIR-1 KD supernatants. **N.** Active TGF-β1 secreted by LAIR-1 WT or LAIR-1 KD cells *in vitro*. **O.** Fold change *in vitro* gene expression of shedding metallopeptidases ADAM17 and ADAM10 in LAIR-1 WT and LAIR-1 KD cells by qPCR, relative to 18s expression. **P-Q.** ADAM10/17 endonuclease targets released *in vitro* in LAIR-1 WT and LAIR-1 KD cell supernatants: **P.** CXCL16 and CX3CL1. **Q.** TNF-α and soluble CD40. Bar graphs show mean and SEM. Unpaired t test. *p<0.05, **p<0.01, ***p<0.001, ****p<0.0001.

**Figure 4.**
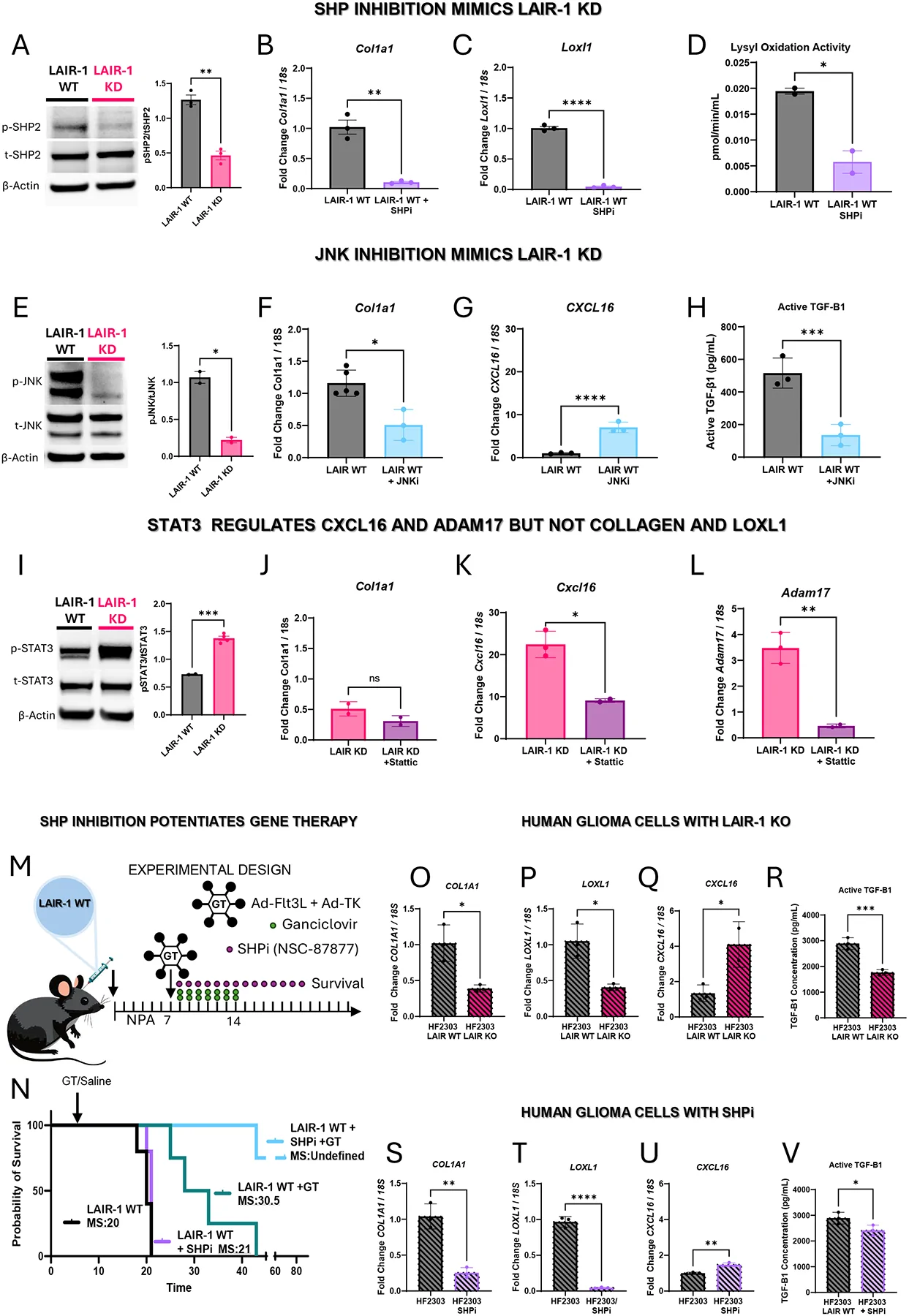
A-D. SHP inhibition mimics LAIR-1 KD. **A.** Quantification of phosphorylation ratio (phosphorylated/ total) of SHP2 in LAIR-1 WT and LAIR-1 KD cell lysates determined by Western blot. Loading control: β-Actin. **B-C.** Fold change of *Col1a1* **(B)** and *Loxl1* **(C)** *in vitro* gene expression of LAIR-1 WT and LAIR-1 WT cells treated with SHP2 inhibitor (NSC-87877) (SHPi), by qPCR, relative to 18s expression. **D.** Lysyl oxidation activity in the supernatant of LAIR-1 WT cells treated with SHPi or control. **E-H. JNK inhibition mimics LAIR-1 KD. E.** Quantification of phosphorylation ratio (phosphorylated/ total) of JNK in LAIR-1 WT and LAIR-1 KD cell lysates determined by Western blot. Loading control: β-Actin. **F-G.** Fold change of *Col1a1* **(F)** and *Cxcl16* **(G)** *in vitro* gene expression of LAIR-1 WT and LAIR-1 WT cells treated with JNK inhibitor (SP-600125) (JNKi), by qPCR, relative to 18s expression. **H.** Active TGF-β1 secreted *in vitro* from LAIR-1 WT treated with JNKi. **I-L. STAT3 regulates *Cxcl16* and *Adam17*, but not *Col1a1* or *Loxl1*. I.** Quantification of phosphorylation ratio (phosphorylated/ total) of STAT3 in LAIR-1 WT and LAIR-1 KD cell lysates determined by Western blot. Loading control: β-Actin. **J-L.** Fold change of *Col1a1* **(J)**, *Cxcl16* **(K)** and *Adam17* **(L)** *in vitro* gene expression in LAIR-1 KD, and LAIR-1 KD cells treated with STAT3 inhibitor (Stattic), by qPCR, relative to 18s expression. **M.** Experimental design schematic. Adult C57BL mice were implanted with LAIR-1 WT glioma cells (day 0). When tumors reached 5×10^5^ flux photos/sec animals received GT: i.t. Ad-Flt3L and Ad-TK, or saline control, followed by 7 days of ganciclovir twice daily i.p. From day 7 onwards animals received daily i.p. SHPi or saline for 14 days. **N.** Survival curve of LAIR-1 WT tumors treated with or without GT and with or without SHPi, as described in M. GT increases MS in LAIR-1 WT from 20 to 30.5 days(**p=0.0062), and in LAIR-1 WT + SHPi (●) from 21 days to undefined (●)(**p=0.0069). **O-R. Human glioma cells with LAIR-1 KO. O-Q.** Fold change of COL1A1 **(O)**, LOXL1**(P)**, CXCL16 **(Q)** *in vitro* gene expression of human glioma cells HF2303 WT compared with HF2303 LAIR-1 CRISPR knock out by qPCR, relative to 18s expression. **R.** Active TGF-β1 secreted by HF2303 cell compared with HF2303 LAIR-1 CRISPR knock out. Note that LAIR-1 KO in human HF2303 cells induces similar changes to those seen in mouse glioma cells with LAIR-1 KD. **S-V. Human glioma cells with SHPi. S-U.** Fold change of COL1A1 **(S)**, LOXL1 **(T)**, CXCL16 **(U)** *in vitro* gene expression of human glioma cells HF2303 treated with SHP2 inhibitor (SHPi), by qPCR, relative to 18s expression. **V.** Active TGF-β1 secreted by HF2303 cell treated with SHPi. Note that treatment with SHPi in human HF2303 cells induces similar changes to those seen in mouse glioma cells with LAIR-1 KD. Bar graphs show mean and SEM. Unpaired t test. *p<0.05, **p<0.01, ***p<0.001, ****p<0.0001.

**Figure 5.**
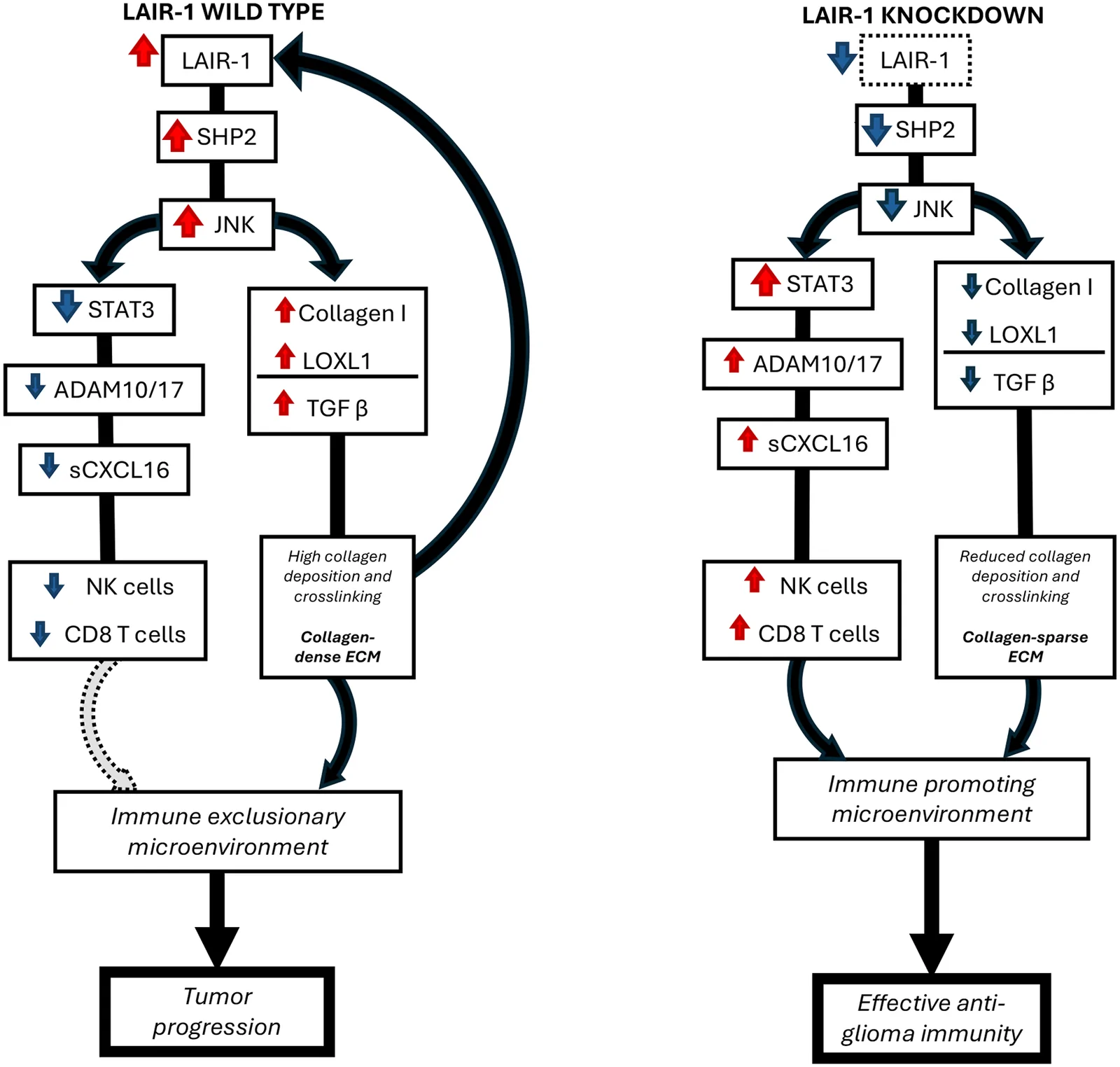
Graphical summary of the downstream changes in LAIR-1 WT glioma cells and in LAIR-1 KD glioma cells. In LAIR-1 WILD TYPE cells (LEFT), LAIR-1 activates SHP2, which activates JNK, resulting in Collagen I, LOXL1 and TGF β production. High collagen deposition and crosslinking performed by LOXL1 generates a collagen-dense extracellular matrix containing high levels of TGFβ, resulting in an immune suppressive microenvironment. High levels of collagen result in a positive feedback that activates LAIR-1 signaling further. Also, SHP2/JNK inactivates STAT3, keeping low levels of AMDA10/17, and therefore low soluble CXCL16. Altogether this stimulates tumor progression, through a dense ECM and immune exclusion. In LAIR-1 KD ells (RIGHT), SHP2, and therefore JNK activity are reduced, causing a lower production of collagen I, LOXL1 and TGFβ. This results in a collagen-sparse extracellular matrix. In parallel, STAT3 becomes active and stimulates ADAM10/17 which release membrane bound CXCL16. Soluble CXCL16 recruits NK and CD8 cells. This results in effective anti-glioma immunity. Thus, this explains how signaling by LAIR-1 in tumor cells causes simultaneously the production of a dense ECM, and immune exclusion.
